# Jellyfish-Inspired Soft Robot Driven by Fluid Electrode Dielectric Organic Robotic Actuators

**DOI:** 10.3389/frobt.2019.00126

**Published:** 2019-11-21

**Authors:** Caleb Christianson, Christopher Bayag, Guorui Li, Saurabh Jadhav, Ayush Giri, Chibuike Agba, Tiefeng Li, Michael T. Tolley

**Affiliations:** ^1^Department of Nanoengineering, University of California, San Diego, La Jolla, CA, United States; ^2^Mechanical and Aerospace Engineering, University of California, San Diego, La Jolla, CA, United States; ^3^School of Aeronautics and Astronautics, Institute of Applied Mechanics, Zhejiang University, Hangzhou, China; ^4^Department of Mechanical Engineering, Howard University, Washington, DC, United States

**Keywords:** dielectric elastomer actuators, artificial muscles, soft robotics, bioinspired robotics, jellyfish swimming

## Abstract

Robots for underwater exploration are typically comprised of rigid materials and driven by propellers or jet thrusters, which consume a significant amount of power. Large power consumption necessitates a sizeable battery, which limits the ability to design a small robot. Propellers and jet thrusters generate considerable noise and vibration, which is counterproductive when studying acoustic signals or studying timid species. Bioinspired soft robots provide an approach for underwater exploration in which the robots are comprised of compliant materials that can better adapt to uncertain environments and take advantage of design elements that have been optimized in nature. In previous work, we demonstrated that frameless DEAs could use fluid electrodes to apply a voltage to the film and that effective locomotion in an eel-inspired robot could be achieved without the need for a rigid frame. However, the robot required an off-board power supply and a non-trivial control signal to achieve propulsion. To develop an untethered soft swimming robot powered by DEAs, we drew inspiration from the jellyfish and attached a ring of frameless DEAs to an inextensible layer to generate a unimorph structure that curves toward the passive side to generate power stroke, and efficiently recovers the original configuration as the robot coasts. This swimming strategy simplified the control system and allowed us to develop a soft robot capable of untethered swimming at an average speed of 3.2 mm/s and a cost of transport of 35. This work demonstrates the feasibility of using DEAs with fluid electrodes for low power, silent operation in underwater environments.

## Introduction

Robots are being used in increasing numbers for underwater exploration and environmental monitoring (Dunbabin and Marques, 2012) and will continue to serve as a valuable data collection tool for scientists (Wynn et al., 2014). However, traditional robots introduce their own risks. Since robots are typically made from rigid materials that can move quickly, they pose inherent danger when operating near fragile structures and creatures and may become lodged in confined spaces. Additionally, underwater robots are usually driven by propellers or jet thrusters, which generate considerable noise and vibration. This additional noise is especially problematic when studying elusive animals or when studying underwater acoustics. Lastly, they consume a large amount of power, requiring considerable batteries or a tether. Recent work by Jaffe et al. demonstrated the deployment of low power profiling floats to study the conditions of large regions of the ocean as they change over time (Jaffe et al., 2017), however the floats only have a buoyancy control and aren't capable of lateral swimming.

The growing field of soft robotics presents a promising approach to designing robots that make them more adaptable to their environments (Calisti et al., 2011; Onal and Rus, 2013; Bauer et al., 2014; Rus and Tolley, 2015; Yuk et al., 2017; Shintake et al., 2018), facilitating safer interactions with fragile objects and creatures, and can enable silent, low power locomotion (Li et al., 2017; Christianson et al., 2018b). Many soft robots are driven either pneumatically or hydraulically by pressurizing and depressurizing fluidic actuators to generate propulsion (Marchese et al., 2014; Katzschmann et al., 2018; Aubin et al., 2019). However, soft robots powered by fluidic actuators require bulky, rigid pumps that consume considerable power.

In this work, we developed a low power, silent, soft, jellyfish-inspired robot driven by fluid electrode dielectric organic robotic actuators (FEDORAs). We used a mechanical model to determine optimal dimensions and validated it with experimental data. Considering fluid dynamic analyses of jellyfish locomotion, we hypothesized that the system would have an optimal driving frequency that maximizes speed and minimizes cost of transport (COT), which we tested experimentally. To enable untethered propulsion, we designed a waterproof, untethered power supply for driving the robot. These efforts resulted in a silent, low-power, untethered soft swimming robot capable of jellyfish-inspired locomotion, comprised of actuators with a maximum deflection of 10 mm, maximum force of 6.1 mN, and maximum work output of 16 μJ. A tethered version swam at a maximum speed of 1.8 mm/s and had a COT of 260. The untethered robot achieved an average speed of 3.2 mm/s with a COT of 35. The layout of the rest of the paper is as follows: in section Background we give an overview of jellyfish locomotion, previous work on jellyfish-inspired robots, and dielectric elastomer actuators; section Robot Design describes the design of our robot; section Experimental Design discusses our experimental design; in section Results we provide our results; and we end with a discussion of the work and our conclusions.

## Background

### Jellyfish Locomotion

In terms of cost of transport (COT), the jellyfish is the most efficient animal at locomotion. COT relates the energy required to move a unit mass a unit distance:

(1) $$\begin{array}{r} COT=\;\frac{E}{mgd}=\;\frac{P}{mgv} \end{array}$$

where *E* is the input energy, *m* is the mass of the animal, *g* is the standard acceleration due to gravity, *d* is the distance traveled, *P* is the input power, and *v* is the animal's velocity. The COT for Aurelia aurita is reported to be between 0.2 and 0.9 (Gemmell et al., 2013). Jellyfish have a thin layer of muscles on the underside of their bell, which they contract to eject a volume of fluid from within their bell. The ejected fluid propels the jellyfish forward. The jellyfish then releases the contraction on their muscles and the elasticity of the jellyfish restores the bell to its initial volume. In other words, the jellyfish primarily expends energy during the contraction phase in which energy not transferred to the fluid can be stored in the elastic structure, and the stored energy can be recovered during the relaxation phase of the swimming cycle. This approach of passive relaxation enables the jellyfish to achieve such high efficiency. Jellyfish swim the fastest and the most efficiently at or near their resonant frequency, based on the stiffness and dimensions of their bell (Hoover and Miller, 2015). The pulse frequency at which jellyfish swim is also inversely proportional to their mass, and they reduce their pulse frequency as they grow to reduce the energetic cost of driving a large mass at a high frequency (McHenry, 2003).

### Actuation for Jellyfish-Inspired Robots

Recently, an untethered jellyfish-inspired soft robot was developed using hydraulic actuators (Frame et al., 2018). One challenge with fluidic actuators is that they necessitate either a pump or piston for pressurizing the chambers, which often draw a considerable amount of power, are most efficient when driven continuously instead of in intermittent pulses, and are made from rigid materials. Nawroth et al. presented a tissue-engineered jellyfish-inspired robot that used cardiac myocytes (heart cells) for actuation (Nawroth et al., 2012). This approach resulted in a completely soft, jellyfish-inspired robot, but led to many fabrication and storage challenges commensurate with implementing biological tissues into a robotic actuator. Several jellyfish-inspired robots have been developed with actuators based on shape memory alloys (SMAs) (Villanueva et al., 2011; Tadesse et al., 2012) and ionic polymer-metal composites (IPMCs) (Yeom and Oh, 2009), but SMAs are challenged by slow response speeds and IPMCs require encapsulation. Ren et al. developed a small scale soft robot that employed an external field to actuate magnetic lappets (Ren et al., 2019). Their work demonstrated the ability to effectively swim and transport cargo, but required an external field for propulsion. Recently, Cheng et al. demonstrated an untethered soft robotic jellyfish that was powered by dielectric elastomer actuators (DEAs) that swam with a maximum speed of 1 cm s^−1^ (Cheng et al., 2019). While the work of Cheng et al. was the first demonstration of an untethered jellyfish-inspired robot powered by DEAs, their work relies upon a prestretched membrane and hydrogel electrodes. Previously, we demonstrated that the performance of soft, swimming robots based on DEAs may be possible with a simpler and more compliant design by implementing prestrain-free membranes and electrodes comprised of a conductive fluid (Christianson et al., 2018a,b).

### Overview of DEAs

DEAs provide an energy efficient method of achieving deformations with high strains for soft robots (Pelrine et al., 2000). Recent efforts on jellyfish-inspired robots driven by dielectric elastomer actuators include those by Godaba et al. in which they developed a pressurized DEA membrane that expanded to eject a volume of water from within a 3D printed shell (Godaba et al., 2016) and a soft, multi-lobed flapping robot developed by Shintake et al. (2016). DEAs are a type of smart material consisting of a dielectric polymer membrane with two conductive electrodes on either side (Carpi et al., 2015). A high voltage is applied through the electrodes across the membrane. As charges accumulate on either side of the membrane, the attraction of opposite charges compresses the film in thickness while repulsion of like charges on the surfaces expands the film in area. If the actuator is laminated to a flexible but inextensible strain-limiting layer, the actuator will curve toward the inextensible layer as it lengthens. One of the benefits of DEAs is that they are energy efficient and have a reported electromechanical conversion efficiency of 90% (Pelrine et al., 2000). Energy loss occurs due to current leakage through the dielectric as well as viscoelastic losses from the material (Chiang Foo et al., 2012). Practically, additional energy is lost when the actuators are discharged, as there are typically energy losses in removing the charges from the actuator (in the worst case they are shunted to ground).

The dielectric film in a DEA is typically either a silicone or acrylic based elastomer (e.g., polydimethylsiloxane—PDMS—or the acrylic adhesive VHB from 3M). The compliant, conductive electrodes can be made from a variety of materials, including thin films of deposited metals; conductive hydrogels; or carbon-based electrodes that are either in a dry powder form, suspended in a silicone grease, or dispersed in an elastomeric matrix. Deposited metals feature high conductivity but their stiffness precludes them from most practical DEA applications and they typically require a deposition in a cleanroom environment or other costly fabrication approaches. Conductive hydrogels are transparent, conductive, and compliant, but they need to be encapsulated to avoid dehydration, impart some non-negligible stiffness, and require some effort to fabricate (Keplinger et al., 2013; Li et al., 2017). Carbon-based electrodes are the most widely used materials for compliant electrodes in DEAs due to their low cost, high compliance, and ease of prototyping, but also need to be encapsulated to prevent smearing under mechanical abrasion. The silicone oil in carbon grease is also reported to disperse through the dielectric membrane, affecting the properties and lifetime of the actuators.

We recently reported that water makes an excellent conductive electrode, especially for underwater applications (Christianson et al., 2017, 2018a,b). Five key advantages of using water for compliant electrodes in a DEA are that fluid electrodes (1) impart no additional stiffness to the structure and are fully compliant to deformations of the actuator; (2) eliminate the need for an external encapsulation layer, reducing the overall stiffness and complexity of fabrication; (3) can be transparent; (4) are inexpensive and straightforward to manufacture; and (5) can be loaded with dyes or other solutions for visual communication or other applications. Previously, we developed an eel-inspired swimming robot that used a series of bimorph fluid electrode dielectric organic robotic actuators (FEDORAs) to undulate through the water at a maximum speed of 1.9 mm/s (Christianson et al., 2018b). In addition, while many DEAs rely upon being prestrained and having a rigid or semi-rigid frame to maintain that strain, prestrained actuators demonstrate a number of disadvantages, including the need to use a rigid frame, the challenge of rupturing the film during prestrain, and the impractability during many applications, especially in soft robotics where compliance is advantageous (Opris, 2018).

## Robot Design

### Description of Overall Design

**Figure 1:**
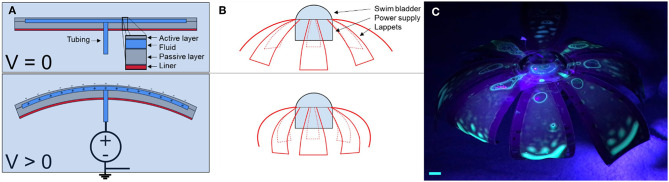
Working principle and fluorescence image of the jellyfish-inspired robot. **(A)** The FEDORAs comprise two elastomeric layers that encapsulate a conductive fluid. When we applied a voltage to the internal fluid electrode w.r.t. the surrounding fluid, Maxwell forces induced a pressure on the dielectric membrane, causing it to lengthen and thin, which resulted in bending toward the inextensible layer. **(B)** To achieve untethered swimming, we designed an axisymmetric array of FEDORAs and attached a waterproof high voltage power supply. A swim bladder provided buoyancy control. When the power supply was on, the electrostatic forces caused the lappets of the robot to bend downward, generating a net thrust upwards. **(C)** Screen captures from Video 3, in which we demonstrated the transparency of the dielectric membrane and fluid electrodes by adding a fluorescent dye to the fluid which we stimulated with an ultraviolet light. Scale bar is 1 cm.

The overall design is based on an axisymmetric array of unimorph actuators, as shown in Figure 1. The outer surface of the robot is an active dielectric elastomer actuator, which expands in area when we apply a voltage to it. To apply the voltage, we implement fluid electrodes—one electrode is a thin film of fluid that is encapsulated underneath the dielectric elastomer, and the other electrode is provided by the surrounding, grounded fluid that the robot is immersed in. A second layer of dielectric elastomer encapsulates the inner fluid electrode. A flexible but inextensible film serves as a strain-limiting layer and provides a small prestrain to the dielectric elastomer. We establish electric contact between the inner fluid electrode and a power supply through a silicone tube with a fluid that provides a conducting path. When we apply a voltage through the fluid across the dielectric elastomer, the elastomer expands in area and the bell of the jellyfish-inspired robot contracts. This contraction accelerates fluid around the edge of the bell, enabling forward propulsion of the robot.

### Design of the Unimorph Actuators

Each actuator comprises four layers—the active dielectric elastomer, the fluid electrode, the passive dielectric elastomer, and the strain-limiting layer, as shown in Figure 1. We designed the unimorph actuator using the optimization results for bending of a multilayer electro-active polymer presented by Balakrisnan et al. (2015), which are based on the tri- and multi-layer analytical models by Benslimane et al. and Devoe et al., respectively (DeVoe and Pisano, 1997; Benslimane et al., 1999). The analytical model predicts the performance of the unimorph actuator as a function of the layer thickness and stiffness of the material. We employed this analytical model to optimize the actuator design for achieving maximum curvature and the desired block force required to produce sufficient thrust underwater. For the unimorph actuator, a large deformation (change in curvature) upon actuation is required to propel the jellyfish by displacing a large quantity of fluid in a single actuation cycle. Additionally, the unimorph actuator is required to generate thrust to overcome the drag generated during each pulse of the actuation cycle. Hence, the blocking force that the unimorph actuator can produce should be sufficient to generate the desired thrust. The unimorph actuator design presented in this paper can be approximated to the trilayer configuration consisting of a mechanically invisible fluid layer at the center.

For generating higher force, the passive layer should have a large relative thickness as compared to the active layer of the unimorph structure. However, a large thickness of the passive layer significantly reduces the change in curvature and deformation of the unimorph structure upon actuation. The minimal thickness and high relative modulus of the passive layer help to achieve maximum curvature in the case of a bilayer structure. Hence, as a compromise between this conflicting relationship for achieving both high force and large deflections, we used a passive layer with matched thickness relative to the active layer and an additional liner with significantly high stiffness that acts as reinforcement for the passive layer. This additional reinforcing layer provides the desired stiffness for the passive layer with minimal change in its thickness and enables us to optimize the design to achieve the desired block force and deformation.

We selected the dielectric elastomer layers from among commercially available materials and electronic components to maximize the strain of actuation. We chose an acrylic-based elastomer for the dielectric layers due to its high actuation response, self-adhesion, and prevalence in DEA research (for comparison with other work). We found four thicknesses for commercially available acrylic elastomers and selected the one that would provide the largest strain at the maximum voltage that we could achieve with a commercially available high voltage power supply (EMCO Q101) without suffering breakdown. We used the analytical mechanical model described by Balakrisnan et al. to determine the optimal thickness of the passive dielectric layer that would maximize work. While a passive layer that matches the thickness of the active layer should achieve the highest deflection, a thicker passive will provide greater force. Thus, we needed to select the thickness that would provide the greatest total work. We also used the same model to predict the increase in work that could be achieved by an additional, stiffer, strain-limiting layer.

**Figure 2:**
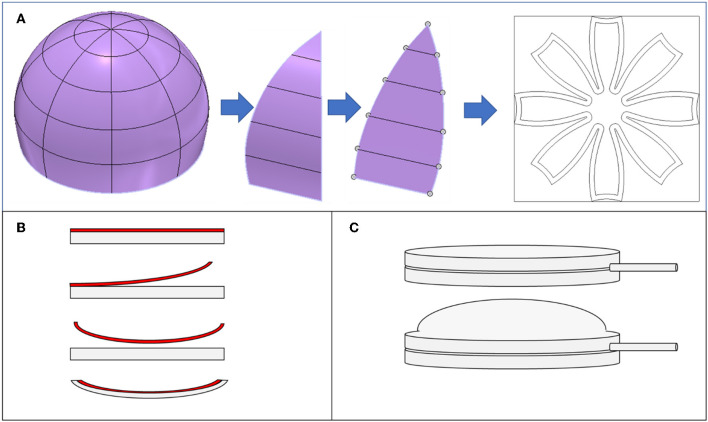
Design of lappets, application of prestrain, and swim bladder. **(A)** Lappets were designed in CAD by segmenting a hemisphere into eight lappets. The lappets were flattened into 2D and then arranged in an axisymmetric array to generate the design for the jellyfish-inspired robot. **(B)** The acrylic elastomer (gray) came affixed to a polyethylene liner (red). When the liner was removed, it exhibited some curvature. When we reapplied the liner to the elastomer, the elastomer was prestrained slightly by the liner and demonstrated a similar curvature. **(C)** The swim bladder was made from two layers of an acrylic elastomer that were adhered to each other at the outer border. A silicone tube was inserted between the layers and pneumatic pressure was applied through the tube to inflate it, providing buoyancy for the jellyfish-inspired robot.

Additionally, DEAs are known to have improved performance when they are prestrained, however, prestrained DEAs typically require a rigid frame to maintain that strain. Alternatively, we imparted the strain-limiting layer with some initial curvature to apply a prestrain to the DEAs (Figure 2B). The acrylic elastomer is shipped on a thin polyethylene liner. When the liner is peeled off of the elastomer, the liner undergoes plastic deformation and exhibits some curvature. When we reapplied the liner with its initial curvature to the elastomer, the layer provided a small initial prestrain to the actuators, which increased their performance.

**Figure 3:**
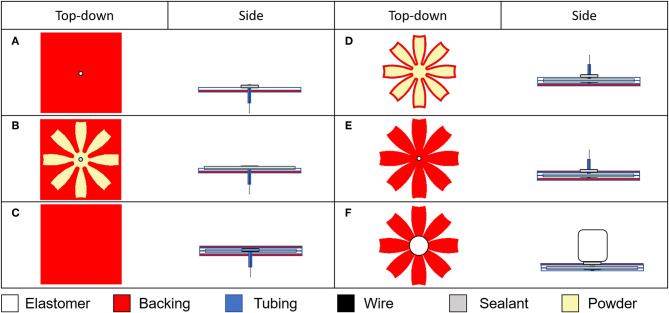
Fabrication process for FEDORA jellyfish. **(A)** We prepared the back layer of the elastomer by cutting it to size and creating a hole in the center of the sheet where we inserted a silicone tube and sealede the tube to the elastomer with a silicone glue. For the untethered case, we threaded a thin, flexible wire through the tubing to reduce the risk of an air in the tube causing an open circuit. **(B)** We used a mask to apply a passivating powder to the lower elastomer layer and then **(C)** encased the powder with the dielectric elastomer layer. **(D)** We removed the backing layer, sealed the underside of the tube with silicone glue, and trimmed the excess material from the actuator through all layers using a laser cutter. After the sealing agent has cured, we injected the fluid electrode through the tubing using a syringe. **(E)** We reapplied the backing layer to the under (tubing) side of the actuator. **(F)** We connected the inner fluid electrode to the driving electronics, removed the backing layer from the dielectric elastomer layer, and applied a passivating agent to the outside of the dielectric elastomer to prevent unintended self-adhesion. For the tethered case, we electrically connected the high voltage power supply to the inner fluid electrode via a metallic syringe tip. For the untethered case, we sealed the tubing shut using a nylon cable tie and electrically connected the power supply to the exposed wire. We then affixed and sealed the electronics to the robot using a silicone glue.

To generate the axisymmetric design for the jellyfish, we designed a hemisphere in CAD with eight segments or lappets. To enable fabrication of a 3D body using laminate fabrication techniques, we flattened the hemisphere onto a planar projection and used layer-by-layer manufacturing to the assemble the structure, as shown in Figure 2. Fabrication details are provided in Materials and Methods and in Figure 3.

### Design of Untethered System

**Figure 4:**
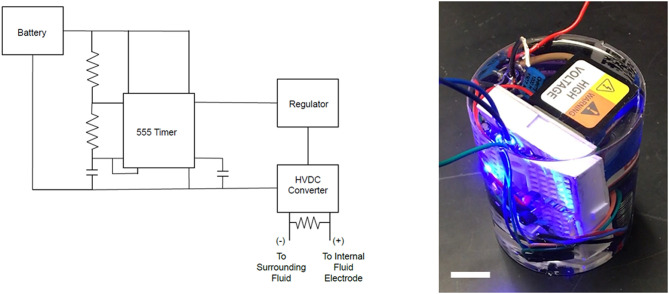
Untethered power supply. **Left**: schematic. A battery powered a 555 timer which we designed to provide a square wave output signal at a desired frequency and duty cycle. We connected the output of the timer to a voltage regulator that provides a consistent maximum voltage to the dc/high voltage dc (HVDC) convertor. The high voltage output of the HVDC convertor was connected to the inner fluid electrode and the ground output is connected to the surrounding fluid. **Right**: photo of power supply encased in silicone with its LED illuminated. Scale bar is 1 cm.

The untethered system needed to provide a high voltage signal to the inner fluid electrode with respect to the surrounding grounded fluid. To accomplish this, we attached a battery to a timing circuit that generated a low voltage square wave signal, as shown in Figure 4. The output of the timer powered a voltage regulator, which then triggered an LED and the high voltage DC/DC convertor. We connected the high voltage output of the HVDC convertor by wire to the internal fluid electrode and immersed the low voltage lead in the surrounding fluid to serve as a ground electrode. In these experiments we used tap water for the fluid electrodes, which we previously found to be sufficiently conductive for actuation (Christianson et al., 2018a,b). We placed a discharge resistor with a high impedance across the high voltage and ground leads to enable passive recovery of the actuators. Since the discharge resistor was in parallel with the actuator and power supply, a low impedance resistor would have reduced the discharge time while a high impedance resistor reduced the power loss, through the resistor during charging. For a power supply with a fixed output power, a higher impedance discharge resistor connected in parallel with the actuator results in a higher operating output voltage across the actuator.

To provide buoyancy control, we assembled a swim bladder out of two layers of an acrylic elastomer using a similar fabrication approach as what was used for the actuators (see materials and methods). A tube was inserted in between the elastomer layers and we injected air with a syringe through a small tube to pressurize the swim bladder. The swim bladder was affixed to the upper surface of the jellyfish to provide buoyancy control. This configuration also has the benefit of reducing the risk of pull-in instability due to local deformation of the high voltage wire underneath.

## Experimental Design

### Measure Deflection and Force as a Function of Frequency for Different Actuator Configurations

**Figure 5:**
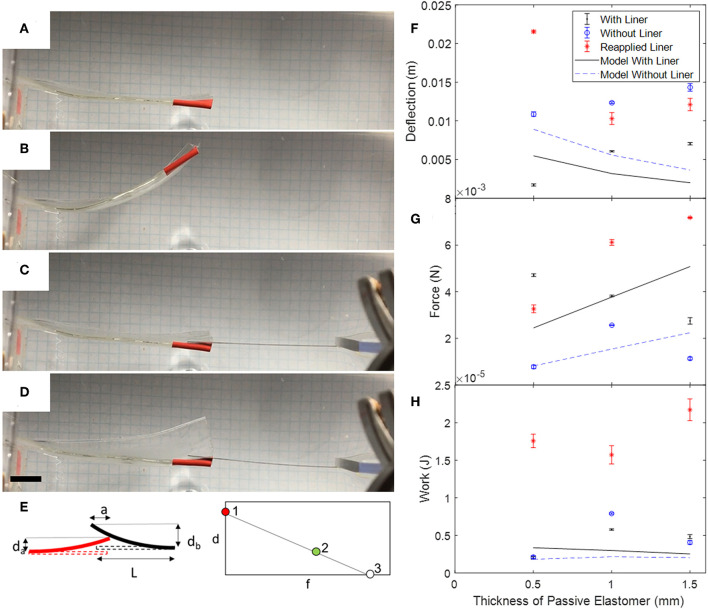
Static tests of unimorph FEDORAs to determine deflection, force, and work as a function of the thickness of the passive layer. Left column: deflection was taken as the difference of the position of the tip when the voltage was off **(A)** and on **(B)**. Tape (red) was applied to the tip as a visual marker to help with tracking. To measure blocked force, we placed a beam of spring steel with length L next to the tip of the actuator **(C)** and measured the deflection of the beam (d_b_) and actuator (d_a_) when the voltage is on **(D)** as shown in the schematic **(E)**. The beam overlapped the tip of the actuator a distance a. We used Euler-Bernoulli beam theory to calculate the force that the actuator imparted on the beam (Equation 2). To estimate the blocked force, we plotted the displacement at no force (1) and the calculated force at a small displacement (d_a_, 2) and assumed a linear relation between the force and displacement and extrapolated to determine the blocked force (3). Right column: comparison of analytical model and experimental results for **(F)** deflection, **(G)** blocked force, and **(H)** work as a function of the thickness of the elastomeric passive layer. Error bars are the standard deviation from three actuations for each actuator configuration. Scale bar is 1 cm.

To determine optimal configurations for the actuators, we measured their maximum deflection and blocked force, as shown in Figure 5. Using the same lateral geometry for each actuator (50.8 mm in width, 76.2 mm in length, with a passive border of 8 mm), we tested actuators with a passive layer thickness of 0.5, 1, and 1.5 mm. We tested the impact of an inextensible strain-limiting layer (the polyethylene backing that the acrylic elastomer is initially affixed to) on actuation. Each test begins with the liner applied (as supplied by the manufacturer), then we measure the actuator again after removing the liner. The acrylic elastomer was rolled up in a tube. When we remove the liner from the elastomer, the liner exhibits a curvature in the opposite direction as the curvature it had when it was on the tube. When we re-apply the liner to the elastomer, the elastomer is prestrained and curls slightly due to the curvature of the liner (Figure 2). We suspended the actuators in water and applied a constant, fixed voltage (7 kV) and measured the maximum deflection. To determine the maximum blocked force, we placed a strip of spring steel with known dimensions adjacent to the actuator and measured the deflection of both the spring steel and actuator when we applied a voltage. Using Euler-Bernoulli beam theory, we calculated the corresponding force at this actuator displacement:

(2) $$\begin{array}{r} F=\;\frac{6yEI}{\left(3l-a\right)a^{2}} \end{array}$$

where *y* is the deflection at the tip of a beam with length *l*, *EI* is the flexural rigidity of the beam, and *a* is the position along the length of the beam where the force was applied. Assuming a linear relationship between force and displacement, we extrapolated to find the blocked force. The area under the curve is calculated as the work output of the actuator. We then compared the work output for three thicknesses of passive layer (0.5, 1, and 1.5 mm) and three conditions of the inextensible backing (backing on, backing removed, and backing reapplied).

### Measure Speed for Tethered Design

To determine the maximum speed as a function of frequency, we attached the robot to a high voltage power supply and drove it at a fixed frequency. The robot was suspended by its silicone tubing which was affixed to a float to maintain a constant vertical position. A wire connected the fluid electrode to the high voltage electronics. Overhead video provided a record of the lateral position of the robot with respect to time and the videos were analyzed using Tracker (Brown, 2019).

### Estimated Power Consumption and Cost of Transport

To estimate the mechanical power required to deform the actuator, we followed an approach described previously (Christianson et al., 2018b) which calculates the electrical input power to be P_electrical_ = C_act_V^2^*f*, based on the capacitance when the actuator is actuated (C_act_), the applied voltage (V), and driving frequency (*f*). To find C_act_, we measured the capacitance of the actuator in the rest state directly using an LCR meter and calculated the expected value of C_act_ based on the change in area and thickness that is predicted from Pelrine's equation and Hooke's law (Pelrine et al., 2000). To calculate the maximum input power as a “worst-case” value for cost of transport calculations, we used the maximum output power of the high voltage direct current (HVDC) power converters, which provide 0.5 watts. At a duty cycle of 50%, the average electrical power from the HVDC convertors to the actuators was 0.25 W. We then calculated the cost of transport using Equation (1) (Paschal et al., 2017).

### Measure Speed for Untethered Swimming

To determine the ability of the robot to swim untethered, we sealed the wireless power supply to the robot and attached a pneumatic swim bladder to the top of it. We tuned the bladder so that the overall structure was slightly negatively buoyant, placed the robot at the bottom of a tank of water, and actuated it at a fixed frequency. We used video to record the position of the robot with respect to time as the robot swam to the top of the tank of water.

## Results

### Deflection, Blocked Force, and Work of Unimorph Actuators

As the thickness of the passive layer increased from 0.5 to 1.5 mm, the deflection also increased (Figure 5). For each thickness of passive layer, the deflection increased when the liner was removed. When the liner was reapplied, the deflection decreased, except in the case when the passive layer was the same thickness as the active layer. The model predicted that the deflection would be greater for the actuator without a liner vs. with a liner, and that the deflection would decrease as the thickness of the passive layer increased. The greatest deflection that we measured was 2.2 cm for the actuator with 0.5 mm thick active and passive elastomer layers and the reapplied liner.

When the actuators were tested with the liner still affixed, the blocked force decreased as the thickness of the passive layer increased. The model predicted that the actuators with the liner would have a greater blocked force than the actuators without a liner and that the blocked force increased as the thickness of the passive layer increased. The maximum blocked force was 7.2 mN for the 0.5/1.5 mm layers and the reapplied liner.

For actuators without an inextensible layer, the model predicted that a peak value of work exists when the passive layer is twice that of the active layer. In contrast, when there was an inextensible layer, the work decreased as the thickness of the passive layer increased. The experimental results for the two cases of the as-prepared liner and actuator without liner agreed with the trend predicted by the model for the case without a liner. However, the actuators with reapplied liners showed the opposite trend. We calculated the maximum work to be 22 μW for the 0.5/1.5 mm layers and reapplied liner.

### Tethered Swimming Performance

**Figure 6:**
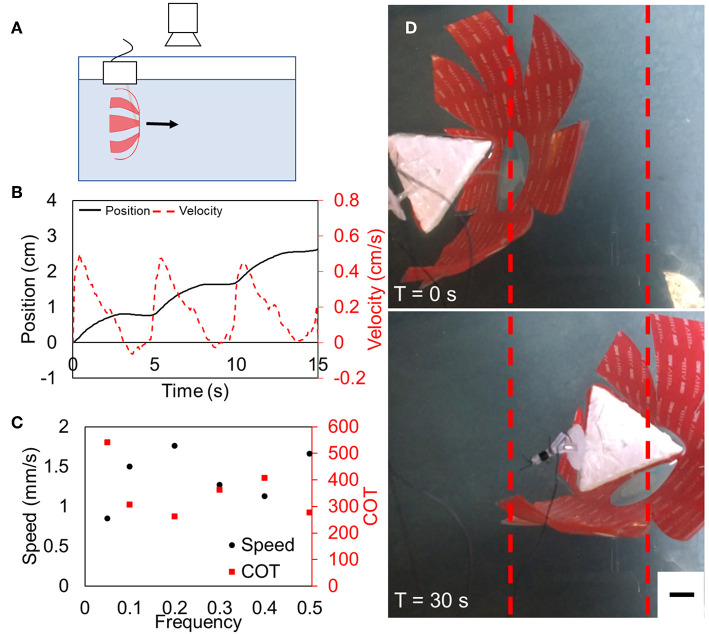
Tethered FEDORA jellyfish swimming results. **(A)** Schematic of experimental setup. **(B)** Representative example of position and velocity as a function of time for three actuation cycles. **(C)** Speed and cost of transport as functions of frequency, demonstrating a maximum speed of 1.8 mm/s and a corresponding COT of 260. **(D)** Still frames taken from swimming tests at the beginning of the test (top) and after 30 s (bottom). Scale bar is 1 cm.

The motion of the robot was approximately sinusoidal with propulsion and coast phases, which we found to be qualitatively similar to the motion of jellyfish. Representative tethered swimming is shown in Video 1. The maximum average speed of the tethered robot was 1.8 mm/s at a driving frequency of 0.2 Hz, as shown in Figure 6, with an instantaneous peak speed of 5 mm/s during contraction. The electrical input power P at 0.2 Hz was 0.25 W and corresponding COT was 260 (Table 1). We measured the swimming speed and calculated the COT for actuation frequencies between 0.05 and 0.5 Hz. While the results shown in Figure 6C suggest that there might be a second harmonic and additional extrema in speed and COT at a driving frequency above 0.5 Hz, empirical results demonstrated that the peak swimming speed was below 0.5 Hz and a thorough investigation of performance at higher frequencies is reserved for future work.

**Table 1:** COT comparison for tethered and untethered swimming.

	**Average input power (W)**	**Mass (kg)**	**Average speed (mm/s)**	**COT**
Tethered	0.25	0.055	1.8	260
Untethered	0.25	0.23	3.2	35

### Untethered Swimming Performance

**Figure 7:**
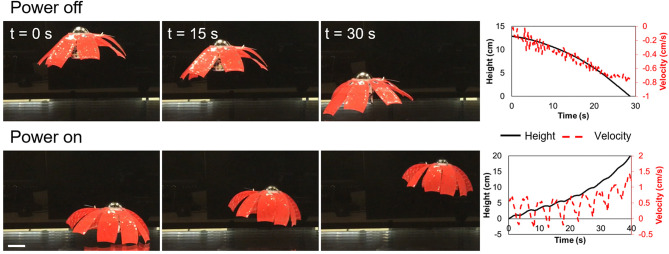
FEDORA jellyfish free-swimming results. **Top row**: unpowered robot was negatively buoyant. **Bottom row**: robot swimming upward with a driving frequency of 0.2 Hz. Right: height from the bottom of the tank and velocity in the vertical direction as a function of time for both the falling (top) and swimming (bottom) cases. Scale bar is 5 cm.

The untethered jellyfish-inspired robot was driven at 0.2 Hz and the average speed over the first three cycles was 3.2 mm/s, as shown in Figure 7 and Video 2, and the peak instantaneous speed was 7.1 mm/s. For a bell margin diameter of 16.3 cm, this corresponds to an average swimming speed of 0.02 BL/s. After three cycles, the upward velocity increased which was likely coupled with a positive change in buoyancy as the pressure on the swim bladder decreased. The COT for the untethered jellyfish was 35, due to the increase in velocity and mass of the robot compared to the tethered case. For this test, we used a battery with a capacity of 180 mAh. The HVDC convertor consumed an average of 0.25 W to power the FEDORAs. At an average power input of 0.25 W and an average speed of 3.2 mm/s, we estimate that a 180 mAh battery would provide enough power for 2.7 h of actuation.

## Discussion

As discussed in section Design of the Unimorph Actuators, the model predicts maximum deflection for a unimorph actuator that behaves as a simple bilayer—i.e., for the case where both layers have the same thickness and modulus. However, when we use FEDORAs without a liner, both dielectric elastomer layers (top and bottom) should experience some strain, proportional to their thickness. In the case where the passive layer is thicker than the active layer, then the strain in the active layer should dominate. Interestingly, we still observed deflection for the case when both layers had the same thickness. This result may be due to our fabrication approach in which a passivating powder is applied to the passive layer which imparts some additional stiffness in the thickness direction of the passive layer, imparting the asymmetry necessary for bending.

As the model is unable to account for prestrain, we were unable to use it to predict the performance of the actuator when we reapplied the liner. The model successfully predicted that the deflection would be greater for an actuator without an inextensible layer and that the force would be greater with the inextensible layer. We also observed the trend that the work of the actuator without a liner would be the greatest when the thickness of the passive layer was twice that of the active layer. However, the trends of how the force and deflection scale with the thickness of the passive layer disagreed with our experimental results. This may be due to the model not taking Maxwell stress on the passive elastomer into account or that there is additional prestrain in the device due to the manufacturing process.

We used the unimorph results to guide our design of the jellyfish-inspired robot. We selected the combination of layer thicknesses that would result in maximizing work for a fully elastomeric robot (0.5 mm thick DEA and 1 mm thick passive layer). To further increase the work, we also used the reapplied liner. Our experimental unimorph results suggest that we may be able to further increase the actuation of our jellyfish-inspired robot by using a thicker passive elastomer layer, but a full comparison between the performance of robots with different thicknesses of the passive layer is reserved for future work.

One challenge with this approach is that prestrain-free DEAs are incapable of providing large forces and thus high-speed propulsion. The maximum tethered and untethered speeds that we measured were 1.8 and 3.2 mm/s, respectively, which may be insufficient for applications in high flow, high speed environments. The speed of our untethered jellyfish is ~3× slower than that of jellyfish of similar mass and the COT of our untethered jellyfish was ~10× greater than that of a jellyfish with a similar mass (Gemmell et al., 2013). The development and employment of sensors, programmability, and lateral control is reserved for future work.

## Conclusion

In this work, we developed a completely soft jellyfish-inspired robot in which all the components—aside from the driving electronics—were flexible or stretchable. The high voltage electrode is encapsulated within a dielectric membrane, ensuring that it is safe for use around animals. As with our previous work using fluid electrodes (Christianson et al., 2018a,b), the FEDORAs actuate completely silently as opposed to traditional ROVs that rely on propeller or jet propulsion, enabling stealthy locomotion. We demonstrate that a flexible but inextensible layer can apply a non-negligible prestrain for DEAs, creasing the performance of the actuators. Many DEAs employ prestrain to improve their performance, but this approach typically requires a rigid frame to maintain that prestrain during actuation. By using an inextensible layer with an initial curvature, we were able to more than double the work output of our actuators without employing a rigid frame. Additionally, we developed a waterproof power supply and demonstrated untethered swimming of our jellyfish-inspired robot. The onboard power supply obviates the need for a tether or external magnetic or electric field. This proof-of-concept prototype demonstrates the feasibility of using FEDORAs for driving bioinspired swimming robots for underwater applications where low power and silent locomotion are important.

## Materials and Methods

We used VHB 4905 (3M) for the 0.5 mm thick dielectric elastomers, VHB 4910 for the 1 mm thick elastomers, and a laminate of the two for 1.5 mm thick layers. Caster sugar was used as the passivating powder and dish soap was used as a passivating liquid. An EMCO Q101 high voltage power convertor was used as a voltage amplifier in all experiments.

## Data Availability Statement

The data generated for this study is available upon request.

## Author Contributions

CC and MT conceived of the project and wrote the initial draft of the paper. CC, CB, GL, AG, and CA fabricated the devices and performed the experiments. CC analyzed the data. SJ helped with modeling and design. TL and MT provided guidance for the project. All authors helped with editing the paper.

### Conflict of Interest

The authors declare that the research was conducted in the absence of any commercial or financial relationships that could be construed as a potential conflict of interest.
